# Synthesis and Properties of Size-Adjustable CsPbBr_3_ Nanosheets for Potential Photocatalysis

**DOI:** 10.3390/ma17112563

**Published:** 2024-05-27

**Authors:** Qi Liu, Hang Li, Xiaoqian Wang, Jiazhen He, Xuemin Luo, Mingwei Wang, Jinfeng Liu, Yong Liu

**Affiliations:** State Key Laboratory of Advanced Technology for Materials Synthesis and Processing, International School of Materials Science and Engineering (ISMSE), Wuhan University of Technology, Wuhan 430070, China; liuq@whut.edu.cn (Q.L.); leehang@whut.edu.cn (H.L.); 303568@whut.edu.cn (X.W.); jizhenhe0606@163.com (J.H.); luoxuemin1123@163.com (X.L.); wmw1842591883@163.com (M.W.); liujinf990528@whut.edu.cn (J.L.)

**Keywords:** CsPbBr_3_ NSs, quantum confinement effect, size-adjustable, photocatalysis

## Abstract

Amidst the rapid advancements in the fields of photovoltaics and optoelectronic devices, CsPbBr_3_ nanosheets (NSs) have emerged as a focal point of research due to their exceptional optical and electronic properties. This work explores the application potential of CsPbBr_3_ NSs in photonic and catalytic domains. Utilizing an optimized hot-injection method and a ZnBr_2_-assisted in situ passivation strategy, we successfully synthesized CsPbBr_3_ NSs with controlled dimensions and optical characteristics. Comprehensive characterization revealed that the nucleation environment and thickness significantly influenced the structure and optical performance of the materials. The results indicate that the optimized synthesis method enables control over the lateral dimensions of the nanoparticles, ranging from 9.1 ± 0.06 nm to 334.5 ± 4.40 nm, facilitating the tuning of the excitation wavelength from 460 nm (blue) to 510 nm (green). Further analyses involving photoresponse and electrochemical impedance spectroscopy demonstrated the substantial potential of these NSs in applications such as photocatalysis and energy conversion.

## 1. Introduction

In recent decades, the rapid development of nanotechnology has enabled the design and fabrication of materials at lower dimensions, uncovering a plethora of novel nanomaterials with unique properties unachievable in traditional bulk or powdered forms. As a rising star among semiconductor nanomaterials, CsPbBr_3_ NSs have garnered global research interest due to their high photoluminescence quantum yield (PLQY), efficient photo-induced charge transfer, tunable spectral continuity, high color purity, and straightforward synthesis methods [1,2,3,4]. CsPbBr_3_ has made significant scientific progress in the fields of solar cells [5,6,7], light-emitting diodes [8,9], display technologies [10,11], and lasers [4,12,13]. These achievements have not only driven the rapid development of related optoelectronic performance and materials research but, compared to other common materials used for catalysis (Table 1 lists the advantages and disadvantages of common electrocatalytic/photocatalytic materials), perovskite structures also exhibit a higher tolerance for defects. Additionally, their band gap can be adjusted by changing their composition and structure, enabling them to absorb light of various wavelengths. This also indicates that CsPbBr_3_ nanocrystals are promising candidates with high photocatalytic potential [14,15,16,17].

The generic structural formula for three-dimensional metal halide perovskites (MHPs) is ABX_3_, where the B-site cations and X-site anions are ionically bonded to form octahedral structures [BX_6_]^4−^ [18]. Two-dimensional (2D) perovskite NSs maintain the corner-sharing connections of [PbX_6_]^4−^ octahedra but are dimensionally confined below the quantum confinement size in the Z-direction, typically consisting of just a few octahedral layers. From an electronic transport perspective, 2D materials exhibit excellent conductivity and are not quantum-confined in certain directions, forming plane-wave-like electronic states in-plane. Optically, zero-dimensional perovskite nanomaterials have a smaller density of states compared to 2D NSs, which can inject more electrons and carriers [19,20,21]. Precisely controlling the number of [BX_6_]^4−^ octahedral layers in the Z-direction, and thus governing the one-dimensional or multi-dimensional growth of CsPbBr_3_ perovskite crystals during fabrication, enables the production of 2D MHPs with varying layer thicknesses. These NSs, smaller than the exciton Bohr radius of CsPbBr_3_ crystals (7 nm), allow for precise control over their optoelectronic properties and achieve blue light emission [22,23]. For example, the fluorescence peaks of CsPbBr_3_ NSs, ranging from one to five mono-layers (MLs), are located at 405, 435, 462, 477, and 488 nm [24], demonstrating a noticeable blue shift as the number of layers decreases, which is a result of the strong quantum confinement effect. The bandgap of 2D CsPbBr_3_ NSs increases as the thickness decreases. Given this tunable inter-layer structure and thickness, along with their enhanced carrier transport capabilities, 2D CsPbBr_3_ NSs have broad potential applications in the catalytic domain.

Due to the quantum confinement effect, high-performance pure-blue-light-emitting CsPbBr_3_ NSs have become a favored blue-light material. Compared to traditional semiconductor quantum dots and rare-earth-doped fluorescent materials, CsPbBr_3_ NSs exhibit many superior optical properties such as a larger absorption cross section, high defect tolerance, high PLQY, high exciton-binding energy, and excellent charge transfer performance [25]. As research into CsPbBr_3_ NSs has deepened, numerous synthesis and control techniques have emerged. For instance, Hu et al. [26] coated ITO onto a glass substrate to form patterned electrodes, then grew CsPbBr_3_ NSs directly on the ITO glass electrodes using chemical vapor deposition, involving processes such as planar printing and etching. Manna et al. [27] successfully increased the lateral size of CsPbBr_3_ NSs to the micrometer level by adjusting the ratio of long-chain to short-chain ligands in the hot-injection method. Moreover, due to the presence of organic ligands on the surface of perovskite NSs and their large surface-to-volume ratio, solution-processed CsPbBr_3_ NSs typically exhibit self-assembly behavior after solvent evaporation. Akkerman et al. [28] proposed controlling the thickness of CsPbBr_3_ NSs by adjusting the amount of HBr added, as the addition of HBr enhances the acidity of the solution, promoting the protonation of OAm and thus forming OAm^+^. The strong interaction of OAm^+^ with the NS surface helps effectively passivate it, inhibiting vertical growth.

Although CsPbBr_3_ NSs exhibit outstanding photophysical properties, stability issues pose significant barriers to their further development. Due to their intrinsic ionic structure and high surface energy, CsPbBr_3_ NSs rely on surface ligands for stabilization. These organic ligands do not bind tightly to the NS surface but instead form a dynamic adsorption–desorption equilibrium. These ligands are prone to detaching during long-term storage or contact with polar solvents, leading to the disintegration of the NSs. In optical device applications, elevated temperatures induce lattice vibrations, increasing the number of thermally active phonons. The coupling of excited-state electrons with these phonons through non-radiative transitions returns them to the ground state, thus reducing their PL intensity. In potential catalytic applications, the photocatalytic efficiency of the material largely depends on its ability to absorb the excitation source, such as radiation. The large surface area of the NSs provides more regions for absorption, stimulating more electron–hole pairs to participate in chemical reactions, and the effective separation of these electron–hole pairs is key to enhancing photocatalytic efficiency. The 2D structure of NSs has a high surface area, and thinner NSs have a larger surface area, exposing more atoms on the surface. If the surface is not fully passivated, numerous surface defects will occur, directly affecting the optoelectronic performance of the NSs. Thus, researchers have attempted to use various types of ligands to passivate CsPbBr_3_ NSs, enhancing the binding force between the ligands and the NSs, reducing the detachment of ligands and surface ions, preventing crystal aggregation into larger particles, stabilizing the colloidal dispersion, or adjusting the crystal structure and morphology. For example, Zhao et al. [29] synthesized blue CsPbBr_3_ NSs of a controllable thickness using tryptophan. The amino and carboxyl groups in tryptophan formed stable complexes with the surface Pb^2+^, not only restricting the vertical growth of NPLs but also enabling the controllable growth of NSs to a certain thickness, significantly enhancing their stability. Feldmann et al. [30] post-treated CsPbBr_3_ NSs with PbBr_2_ to replenish Pb and Br vacancies on the NS surface, significantly enhancing the PL intensity of the passivated samples.

As research into halide perovskite materials deepens, their potential in photonic conversion and catalytic applications gradually emerges. CsPbBr_3_ NSs, with their unique electronic structure and optical properties, have become a research hotspot. However, maintaining their optoelectronic performance while enhancing their structural stability remains a major challenge. This work innovatively creates a bromide ion-rich environment based on the conventional hot-injection method, enabling the tuning of the size of CsPbBr_3_ NSs by simply adjusting the concentrations of OctAm and OctAc. This approach synthesizes highly uniform CsPbBr_3_ NSs and achieves a strong confinement of pure CsPbBr_3_ perovskite with blue emission [31,32]. Optical and electrochemical testing of the CsPbBr_3_ NSs indicate that these synthesized NSs have potential applications in the field of photocatalysis.

## 2. Materials and Methods

### 2.1. Materials

Lead (II) bromide (PbBr_2_, 99.999%), cesium carbonate (Cs_2_CO_3_, 99.9%), zinc bromide (ZnBr_2_, Aladdin, 99%), 1-octadecene (ODE, 90%), oleic acid (OA, 90%), oleylamine (OAm, 70%), octanoic acid (OctAc, 99%), octylamine (OctAm 99,5%), and hexane (95%) were purchased from Aladdin. All chemicals were used without any further purification.

### 2.2. Methods

#### 2.2.1. Preparation of Cs–Oleate Precursors

A 0.064 g amount of Cs_2_CO_3_ and 20 mL of OA were loaded into a 50 mL 3-neck flask, dried for 1 h at 120 °C under vacuum, and then heated under N_2_ to 140 °C until all of the Cs_2_CO_3_ had reacted with the OA.

#### 2.2.2. Synthesis of CsPbBr_3_ NSs

First, 10 mL ODE, 0.17 g PbBr_2_, and 0.17 g ZnBr_2_ were loaded into a 3-neck flask and degassed under vacuum at 120 °C for 25 min. Then, 0.5 mL OA, 0.25 mL OAm, and a proper volume of OctAm and OctAc (e.g., 0.4 mL to obtain NSs with a transverse size of about 47 nm; see main text for details) were injected at 120 °C under N_2_. The solution was degassed under vacuum at 120 °C for 2 min and then the temperature was raised to 150 °C. After complete solubilization of the PbBr_2_, 0.1 mL of the Cs–oleate precursors was quickly injected. After 45 min, the reaction was stopped by rapidly cooling the solution down to room temperature in an ice-water bath.

#### 2.2.3. Isolation and Purification of Crystals

The solution was transferred to a 50 mL centrifuge tube, to which 10 mL of hexane was added. The mixture was then centrifuged at 8000 rpm for 5 min. The centrifuge used in this work was the TG16-WS model from Changsha Dongwang Experimental Instrument Co., Ltd. (Changsha, China). The supernatant was discarded, and the precipitate was redispersed in 5 mL of hexane. This suspension was subjected to centrifugation at 500 rpm for 3 min, and the resulting supernatant was collected for further use.

### 2.3. Characterization of Materials

#### 2.3.1. Transmission Electron Microscopy (TEM) Characterization

TEM images were acquired with a JEOL JEM-1400 (Fargo, ND, USA) Plus electron microscope, equipped with a thermionic emission gun, and operated at an acceleration voltage of 120 kV. The NS samples were prepared by depositing a dilute suspension of NSs in hexane onto carbon-coated copper grids. The NSs’ size distribution was determined from the TEM images using Nano Measurer software version 1.02.0005.

#### 2.3.2. X-ray Diffraction (XRD) Characterization

The XRD patterns were recorded using a Bruker D8 ADVANCE diffractometer equipped with a Cu Kα radiation source (λ = 1.540598 Å). Measurements covered the 2θ range from 10° to 35°, with a step size of 0.05° and a dwell time of 0.5 s per step. The X-ray tube was operated at 40 kV and 40 mA. For XRD analysis, the NSs were drop-casted from a concentrated hexane suspension onto SiO_2_/Si substrates. Data interpretation was performed using Jade 6.5 software.

#### 2.3.3. Fourier Transform Infrared (FTIR) Spectroscopy Characterization

FTIR spectra were collected at an ambient temperature using a Nicolet 6700 spectrometer. The spectral acquisition covered a wavenumber range of 1000–4000 cm^−1^ at a resolution of 0.019 cm^−1^ and a scan rate of one scan per second. The instrument’s interferometer operated on the principle of frequency modulation interference, producing an interferometric light pattern that was directed onto the sample. The detected interferometric light, carrying material-specific information, was converted into a spectrum via Fourier transformation using the spectrometer’s software (OMNIC 3.0). For analysis, a dense hexane suspension of CsPbBr_3_ NSs was spread onto a glass slide, dried, and subsequently examined.

#### 2.3.4. High-Resolution Transmission Electron Microscopy (HRTEM) Characterization

The morphology of the perovskite samples was investigated using a Talos F200S field emission HRTEM from Thermo Fisher Scientific (Waltham, MA, USA), operating at an acceleration voltage of 200 kV. Samples were prepared by depositing them onto 200-mesh copper grids backed by a carbon film prior to imaging.

#### 2.3.5. X-ray Photoelectron Spectroscopy (XPS) Characterization

XPS measurements were performed on the perovskite NSs utilizing an AXIS SUPRA spectrometer. This equipment includes a monochromatic Al-Kα X-ray source and a Thermo Scientific Kα detector. The spectral data were formatted to VGD and analyzed using Avantage software version 5.9922. Calibration of the binding energy scale was referenced to the carbon C1s peak at 284.8 eV, corresponding to the C-C bond.

#### 2.3.6. Fluorescence Spectrum Measurements

UV-vis absorption spectra were obtained with a Shimadzu UV-1800 spectrophotometer, and steady-state PL measurements were conducted on a Shimadzu RF-6000 spectrometer. The CsPbBr_3_ NSs were excited at a wavelength of 350 nm. TRPL spectra were acquired using a single-photon counting method. Samples for optical analysis were prepared in quartz cuvettes with a path length of 10 mm.

#### 2.3.7. Thermogravimetric Analysis (TGA) Measurements

A thermogravimetric analysis was conducted using an STA449F3 Jupiter from Netzsch (Selb, Germany). The temperature was ramped up from room temperature to 500 °C at a heating rate of 10 °C/min under a nitrogen atmosphere. Samples were placed in alumina crucibles to avoid any reaction with the container.

#### 2.3.8. Photoelectrochemistry/Electrochemistry Measurements

Photoelectrochemical and electrochemical impedance spectroscopy (EIS) measurements were carried out using a Zennium workstation. The system was set up with a three-electrode configuration comprising the sample as the working electrode, a platinum disk as the counter electrode, and a Ag/AgCl electrode in saturated KCl as the reference (E_Ag/AgCl_ = +0.1989 V vs. NHE). Measurements were conducted in an electrolyte of 0.5 M Na_2_SO_4_. A 405 nm LED served as the light source for photocurrent measurements, while the intensity was verified using a Newport photometer. EIS was performed at a bias of −0.1 V vs. NHE, with a 10 mV amplitude over a frequency range of 100 mHz to 20 kHz.

## 3. Results

**Scheme 1:**
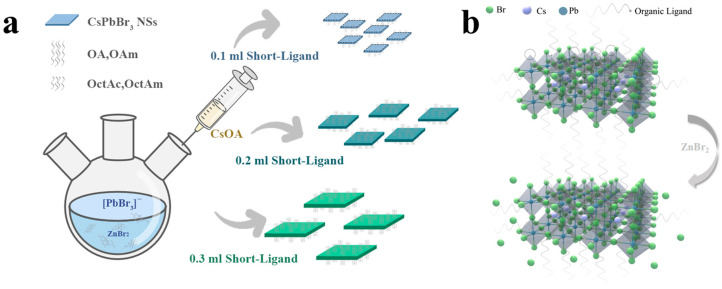
(**a**) Schematic diagram of the synthesis procedure for CsPbBr_3_ NSs. (**b**) Passivation schematic image of CsPbBr_3_ NSs.

CsPbBr_3_ NSs possess a high surface area, significantly impacting their optoelectronic properties due to their surface states. To stabilize the surface of NSs, ligand passivation methods are commonly employed. However, traditional ligands such as OA and OAm exhibit relatively weak interactions with CsPbBr_3_ NSs, which may lead to ligand desorption from the NS surface during washing and purification processes, resulting in numerous surface defects. The use of short-chain ligands facilitates a controlled synthesis of the lateral dimensions of the NSs while maintaining the vertical dimensions under strong quantum confinement [27]. Additionally, the introduction of an excess of Br^−^ ions can effectively passivate the perovskite surface by filling Br^−^ vacancy defects [33]. In a Br-rich reaction system, Pb^2+^ ions initially form [PbBr_3_]^−^ complexes, which subsequently transform into octahedral structures. Once an octahedral structure is established, under the influence of Cs-OA, it provides sufficient nucleation sites that promote rapid crystal nucleation. By adjusting the concentration of short-chain ligands (OctAc, OctAm) as described in Scheme 1a, we can precisely control the lateral dimensions and thickness of the NSs in the Z-direction, thereby influencing their optical properties. Additionally, by adding ZnBr_2_, we create a bromine-rich environment aimed at enhancing the performance of the NSs and reducing surface defects (as illustrated in Scheme 1b). Through this approach, continuous tunable emission from green to blue light can be achieved in the CsPbBr_3_ NSs.

**Figure 1:**
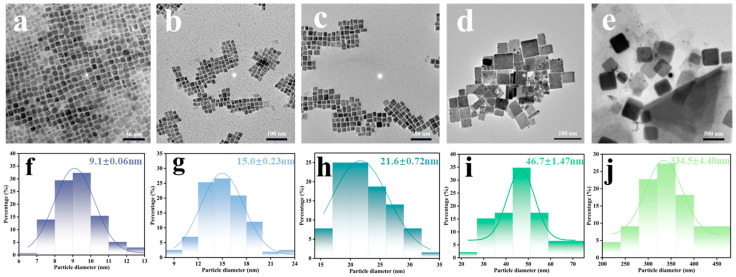
TEM images of CsPbBr_3_ NSs with the addition of (**a**) 0.1 mL, (**b**) 0.2 mL, (**c**) 0.3 mL, (**d**) 0.4 mL, and (**e**) 0.5 mL short-ligand volumes. The size distribution of the NSs was synthesized by adding (**f**) 0.1 mL, (**g**) 0.2 mL, (**h**) 0.3 mL, (**i**) 0.4 mL, and (**j**) 0.5 mL short-ligand volumes.

Figure 1a–e and Figure S1 display the TEM morphology of synthesized CsPbBr_3_ NSs as the amount of short-chain ligand increases (0.1 mL, 0.2 mL, 0.3 mL, 0.4 mL, 0.5 mL, and 0.05 mL). These images reveal the significant impact of ligand concentration on the morphology and size of the NSs. From the particle size distribution graphs in Figure 1f–j the corresponding lateral dimensions are approximately 9.1 ± 0.06 nm, 15.0 ± 0.23 nm, 21.6 ± 0.72 nm, 46.7 ± 1.47 nm, and 334.5 ± 4.40 nm. It should be noted that with 0.05 mL of the short-chain ligand, the morphology of the NSs tends to transition towards quantum dots (Figure S1), and therefore, this size was not statistically analyzed. As the amount of short-chain ligand increases, the lateral dimensions of the CsPbBr_3_ NSs significantly expand, and the rate of the size increase accelerates with the amount of ligand. However, when the ligand amount is reduced, as seen at 0.05 mL, the morphology tends to exhibit quantum dots and aggregation occurs, indicating that too low a ligand concentration is detrimental to the stable formation of NSs, and an appropriate amount of ligand can promote planar growth while preventing the aggregation caused by overly dense nucleation sites. Further comparisons with samples without added ZnBr_2_ in Figure S2 show a poorer morphology and uneven size distribution of the NSs, likely due to the lack of effective bromine vacancy compensation and surface passivation mechanisms during growth, illustrating the significant positive impact of introducing ZnBr_2_ on the morphology and stability of the NSs. The Br-rich environment provided by ZnBr_2_ optimizes the stability of the perovskite structure and the uniformity of its morphology, and reduces the formation of non-uniform nucleation sites. The role of ZnBr_2_ extends beyond just replenishing surface bromine vacancies; it likely passivates the surface of the nanocrystals, reducing the formation of amorphous regions, thereby enhancing crystallinity and morphological integrity, which is crucial for understanding the growth mechanisms of perovskite materials and controlling the optoelectronic properties of nanomaterials. By finely controlling the synthesis conditions, we can not only regulate the size and morphology of these NSs but also further manipulate the material’s optoelectronic performance, which holds significant practical value in developing high-performance optoelectronic devices and catalysts.

**Figure 2:**
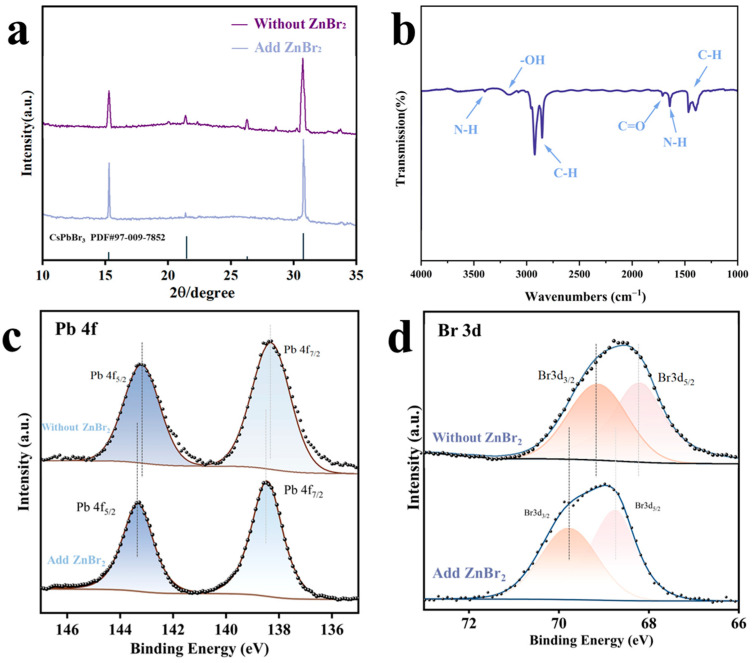
(**a**) XRD patterns of CsPbBr_3_ NSs with and without ZnBr_2_; (**b**) FTIR spectrum of CsPbBr_3_ NSs; (**c**) XPS profiles of CsPbBr_3_ NSs Pb 4f with and without ZnBr_2_; (**d**) XPS profiles of CsPbBr_3_ NSs Br 3d with and without ZnBr_2_. (The dotted line is the middle of the peak).

The XRD patterns (Figure 2a) display the crystallographic differences between 15 nm CsPbBr_3_ NSs synthesized with and without the addition of ZnBr_2_. The main diffraction peaks of both samples, located at 15.1° and 30.4°, correspond to the (100) and (200) crystal planes of cubic-phase CsPbBr_3_ (PDF#97-009-7852), indicating a pronounced preferential orientation along the (100) plane. The HRTEM measurements in Figure S3 show an interplanar spacing of 0.591 nm, consistent with the (100) planes of cubic-phase CsPbBr_3_. Compared to the non-ZnBr_2_-supplemented samples, those with ZnBr_2_ exhibit sharper diffraction peaks in the XRD chart, indicating higher crystallinity and more complete crystal properties, whereas the samples without ZnBr_2_ display additional diffraction peaks from other crystal planes and more stray peaks. The FTIR spectrum in Figure 2b reveals vibrational modes characteristic of surface ligands on the CsPbBr_3_ NSs. The absorption peaks at 3396 cm^−1^ and 1641 cm^−1^ are attributed to the stretching and bending vibrations of the amine N-H bonds, respectively. The peak at 3166 cm^−1^ indicates the presence of carboxylic O-H bonds, corresponding to the stretching vibrations of carboxyl groups. Lower-frequency peaks at 2923 cm^−1^, 2854 cm^−1^, and 1396 cm^−1^ arise from the asymmetric and symmetric stretching vibrations of the carbon chain C-H bonds, respectively. The peak at 1712 cm^−1^ is derived from the stretching vibration of the carboxylic C=O bond, and the peak at 1467 cm^−1^ is assigned to the in-plane bending vibrations of the methylene group’s C-H single bonds. The appearance of different absorption peaks in the FTIR spectrum indicates changes in the vibrational modes of functional groups, demonstrating an effective protonation of oily carboxylic acids and amines on the surface of the CsPbBr_3_ NSs. Figure 2c,d and Figure S4 present analyses of the surface chemical states of the CsPbBr_3_ NSs via XPS. In Figure S4, regardless of the ZnBr_2_ addition, the peaks for Cs 3d_3/2_ and Cs 3d_5/2_ remain stable at 738.0 eV and 724.1 eV, respectively, indicating a consistent chemical environment for Cs in both samples. For samples without any added ZnBr_2_, the binding energies for Pb 4f_5/2_ and Pb 4f_7/2_ are located around 143.2 eV and 138.3 eV, respectively, and Br 3d_3/2_ and Br 3d_5/2_ are at 69.2 eV and 68.2 eV. In contrast, with the ZnBr_2_ addition, these peaks shift to higher binding energies, where Pb 4f_5/2_ and Pb 4f_7/2_ move to 143.4 eV and 138.5 eV, respectively, and Br 3d_3/2_ and Br 3d_5/2_ move to 69.8 eV and 68.8 eV. This shift in binding energy suggests stronger Pb-Br interactions, likely due to the halogen atoms’ lower activation energy facilitating their migration [34,35,36]. On CsPbBr_3_ NS surfaces not passivated with ZnBr_2_, a large number of Br vacancies lead to many incomplete [PbBr_6_]^4−^ octahedra, while the Br-rich environment-synthesized NSs exhibit stronger Pb-Br interactions, showing improved surface structure and enhanced crystallinity, effectively passivating halide vacancy defects on the nanocrystal surface [37,38].

**Figure 3:**
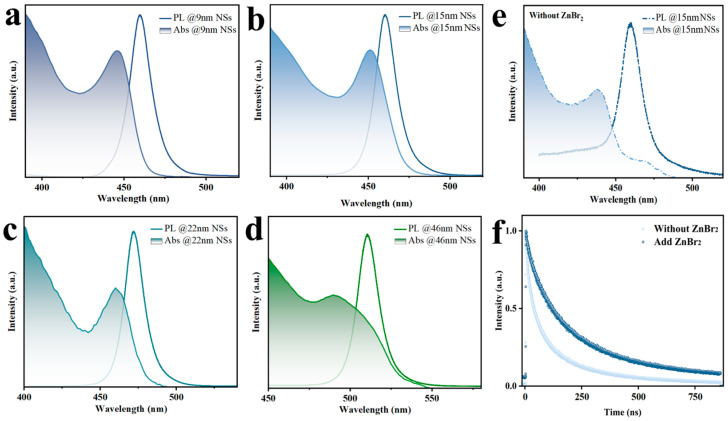
PL and UV-vis spectra of NSs with transverse dimensions of (**a**) 9 nm, (**b**) 15 nm, (**c**) 22 nm (**d**), and 47 nm; (**e**) PL and UV-vis profiles of NSs with a lateral size of 15 nm without ZnBr_2_; (**f**) TRPL curves of NSs with and without ZnBr_2_.

We conducted optical characterization of CsPbBr_3_ NSs with lateral sizes of approximately 9 nm, 15 nm, 22 nm, and 47 nm, including UV-vis absorption spectroscopy and PL spectroscopy analyses. In this experimental setup, once the lateral dimension exceeds 46 nm, the thickness along the Z-axis surpasses the range affected by quantum confinement effects, producing optical properties similar to those of 3D CsPbBr_3_. Hence, the optical performance of the 335 nm sample was not analyzed in this study. Figure 3a–d show the PL emission peaks of NSs with dimensions of 9 nm, 15 nm, 22 nm, and 47 nm at 460 nm, 460 nm, 472 nm, and 510 nm, respectively, while their absorption peaks are located at 446 nm, 451 nm, 460 nm, and 490 nm. As the average lateral size decreases, the thickness of the nanosheets correspondingly diminishes, with the measured average thicknesses of the inorganic layers being approximately 2.35 nm, 3.35 nm, and 11.05 nm, respectively. Figure S5 displays the TEM images of self-assembled NSs with lateral sizes of 15 nm, 22 nm, and 47 nm, facilitated by strong intermolecular forces (since the NSs of 9 nm and 15 nm show the same PL peaks, indicating similar quantum confinement effects due to changes in vertical dimensions, their self-assembled TEM images are not listed). As the thickness reduces to below 7 nm, quantum confinement effects become apparent, causing the electronic energy levels to split into discrete states and leading to a blue shift in the excitation peaks. This indicates that by controlling the amount of short-chain ligands, one can effectively adjust the lateral and vertical dimensions of the NSs, thus manipulating the strength of their quantum confinement state. Further, comparing the PL and UV-vis spectra of 15 nm NSs with and without added ZnBr_2_ (Figure 3b,e), it is found that although the PL peak positions of the non-ZnBr_2_-supplemented samples remained unchanged, the overall quality of the PL spectrum was reduced. Additionally, the Stokes shifts for NSs with and without ZnBr_2_ were 9 nm and 22 nm, respectively, possibly because the passivation treatment in the CsPbBr_3_ NSs weakened the coupling between electronic states and vibrational states, reducing non-radiative relaxation processes and thus making it less likely for electronic transitions to significantly alter molecular vibrational energy levels, which consequently reduced the Stokes shift. The TRPL three-exponential fitting in Figure 3f shows that the average fluorescence lifetimes for NSs with and without added ZnBr_2_ were approximately 12 ns and 22 ns, respectively. Non-passivated NS surfaces have many defects, acting as recombination centers for electron–hole pairs, leading to increased decay channels and thus reducing the fluorescence lifetime [39]. Additionally, to assess the thermal stability of the samples before and after passivation, we conducted a thermogravimetric analysis on the two types of samples. As shown in Figure S6, the non-passivated NSs exhibited a more pronounced weight-loss trend, demonstrating that optimized reaction conditions significantly enhance the thermal stability of NSs. The bandgap tuning of CsPbBr_3_ NSs demonstrates significant potential in the field of photocatalysis. By adjusting the bandgap of CsPbBr_3_ NSs relative to other materials to form heterostructures, it is easy to form Type II band structures, effectively promoting the separation of electrons and holes, thereby significantly enhancing their catalytic performance.

**Figure 4:**
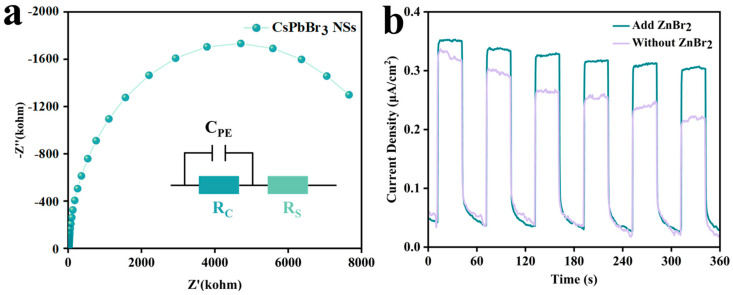
(**a**) EIS results (Nyquist plot) for CsPbBr_3_ NSs with a lateral size of 15 nm. The inset illustrates the equivalent circuit model. (**b**) Instantaneous photocurrent response of CsPbBr_3_ NSs with and without ZnBr_2_ in neutral water (0.5 M Na_2_SO_4_) at −0.1 V vs. NHE.

As depicted in Figure 4, photoresponse current density measurements and EIS analyses were conducted for the 15 nm CsPbBr_3_ NSs. In the equivalent circuit model, CPE represents the double-layer capacitance, RS indicates the solution resistance between the reference and working electrodes, and RC corresponds to the charge transfer resistance at the electrode. The EIS spectra of these CsPbBr_3_ NSs exhibited a characteristic semicircular arc, which is indicative of charge transfer resistance. In order to accurately capture the photocurrent generated upon illumination and to exclude any extrinsic influences, the photocurrent distribution was periodically recorded upon activation of the light source. Notably, the measured photocurrent density was nearly negligible when the light source was switched off but surged instantaneously with reactivation at 30 s intervals. This cyclic diminution of current upon deactivation of the light source every 30 s further corroborates the efficient carrier migration properties of the CsPbBr_3_ perovskite material. Further comparison with NSs not passivated with ZnBr_2_ shows that under the same test conditions, the non-passivated NSs exhibit relatively lower photocurrent density. This is attributed to increased charge recombination rates caused by defects, which in turn leads to reduced carrier mobility. Additionally, we conducted photocurrent density tests on NSs stored at room temperature for one week across four different time periods, as shown in Figure S7. The results indicate that although the photocurrent intensity degraded over time, the NSs still retained a certain level of photoresponse capability after a week. Therefore, CsPbBr_3_ nanosheets with low levels of defects demonstrate potential applicability in the field of photoelectrocatalysis. The substantial surface area of CsPbBr_3_ NSs provides an expansive medium for carrier transport and reaction, thereby augmenting the material’s responsiveness to light. The periodic photocurrent response under illumination conditions reveals the stability and reproducibility of CsPbBr_3_ NSs, laying a foundation for their application in areas such as environmental purification, energy conversion, and sensing.

## 4. Conclusions

This work successfully synthesized CsPbBr_3_ NSs using a hot-injection method with varied ratios of long- and short-chain ligands and a ZnBr_2_-assisted in situ passivation strategy. The structure and optoelectronic properties of these NSs were comprehensively analyzed using a series of characterization techniques. TEM results revealed that the NSs exhibited a uniform and regular morphology with a size distribution ranging from 9.1 ± 0.06 nm to 334.5 ± 4.40 nm, demonstrating that precisely controlling the synthesis conditions can regulate the dimensions of the NSs. XRD and HRTEM analyses further disclosed the high crystallinity of the NSs and their preferential orientation along specific crystal planes, which contributes to optimizing optoelectronic performance. FTIR confirmed the presence of oily carboxylic acids and amines on the surface of the CsPbBr_3_ NSs, playing a significant role in their effective separation and stabilization. XPS tests comparing samples with and without added ZnBr_2_ as reaction conditions validated the effectiveness of the in situ passivation strategy in controlling surface defects and enhancing chemical stability. Our EIS test results suggest a certain efficiency of charge separation when these NSs are combined with other semiconductor materials, indicating their potential application in photocatalysis and photonic conversion. Future work will focus on further optimizing the conditions for synthesizing NSs to enhance their stability and performance in practical applications, particularly exploring composites with other functional materials to develop new high-efficiency optoelectronic devices and catalysts.

## Figures and Tables

**Table 1:** Advantages and disadvantages of recently developed photocatalytic/electrocatalytic materials.

Material Type	Application Areas	Advantages	Disadvantages
Perovskites	Electrocatalysis/photocatalysis	Efficient photocatalysis, adjustable bandgap	Poor stability, potential toxicity
Metals	Electrocatalysis	High conductivity, good activity	Prone to corrosion, expensive
Metal oxides	Electrocatalysis	Good stability and activity	High cost, high overpotentials needed
MXenes	Electrocatalysis/photocatalysis	High conductivity, excellent mechanics	Poor oxidative stability, dispersibility
MOFs	Electrocatalysis	High surface area, diverse functions	Structural instability, complex fabrication
COFs	Electrocatalysis	Designable, precise reaction control	Fabrication difficulty, water instability
Graphene-based materials	Electrocatalysis/photocatalysis	High stability, suitable for electrodes	Limited activity, requires modifications

## Data Availability

Data are contained within the article.

## Supplementary Materials

The following supporting information can be downloaded at https://www.mdpi.com/article/10.3390/ma17112563/s1, Figure S1: TEM image of the sample with a 0.05 mL short-ligand addition; Figure S2: TEM images of CsPbBr_3_ NSs at different short-ligand concentrations without ZnBr_2_; Figure S3: HRTEM images of CsPbBr_3_ NSs; Figure S4: XPS profiles of CsPbBr_3_ NSs Cs 3d (a) with ZnBr_2_ and (b) without ZnBr_2_; Figure S5: The emission peaks of (a) 460 nm, (b) 482 nm, and (c) 510 nm correspond to TEM images of the NSs after self-assembly; Figure S6: TGA profiles of CsPbBr_3_ NSs with and without ZnBr_2_ under N_2_ flow and atmospheric pressure in the temperature range 25–500 °C; Figure S7: Instantaneous photocurrent response of CsPbBr_3_ NSs stored at room temperature for 0, 1, 3, and 7 days in neutral water (0.5 M Na_2_SO_4_) at −0.1 V vs. NHE.
