# Supplementary material for: Synthesis and Properties of Size-Adjustable CsPbBr3 Nanosheets for Potential Photocatalysis
Source: Materials (Basel). 2024 May 27;17(11):2563. doi: 10.3390/ma17112563 (PMC11173759; doi:10.3390/ma17112563)
Supplement: Supplementary file 1 [file materials-17-02563-s001.zip › materials-3000952-supplementary.pdf]

Synthesis and Properties of Size-Adjustable $CsPbBr_3$ Nanosheets for Potential Photocatalysis

Qi Liu, Hang Li, Xiaoqian Wang, Jiazhen He, Xuemin Luo, Mingwei Wang, Jinfeng Liu and Yong Liu *

State Key Laboratory of Advanced Technology for Materials Synthesis and Processing, International School of Materials Science and Engineering (ISMSE), Wuhan University of Technology, Wuhan 430070, China; liuq@whut.edu.cn (Q.L.) leehang@whut.edu.cn (H.L.); 303568@whut.edu.cn (X.W.); jizhenhe0606@163.com (J.H.); luoxuemin1123@163.com (X.L.); wmw1842591883@163.com (M.W.); liujinf990528@whut.edu.cn (J.L.)

* Correspondence: liuyong3873@whut.edu.cn

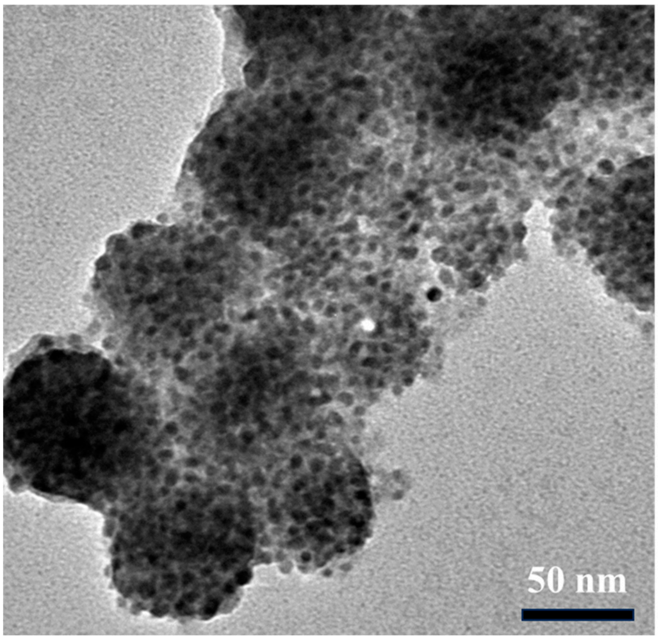

Figure S1. TEM image of the sample with addition of 0.05 ml short ligand

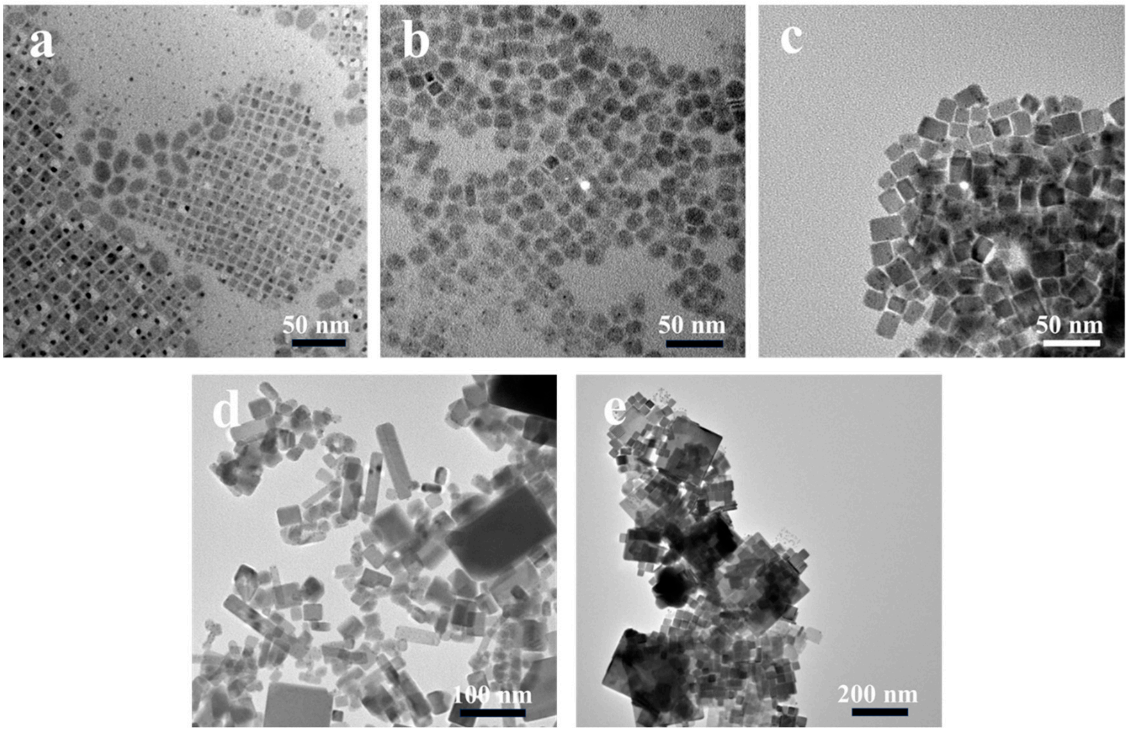

Figure S2. TEM images of $CsPbBr_3$ NSs at the addition of **(a)** 0.1ml, **(b)** 0.2ml, **(c)** 0.3ml, **(d)** 0.4ml and **(e)** 0.5ml short ligand volumes without $ZnBr_2$.

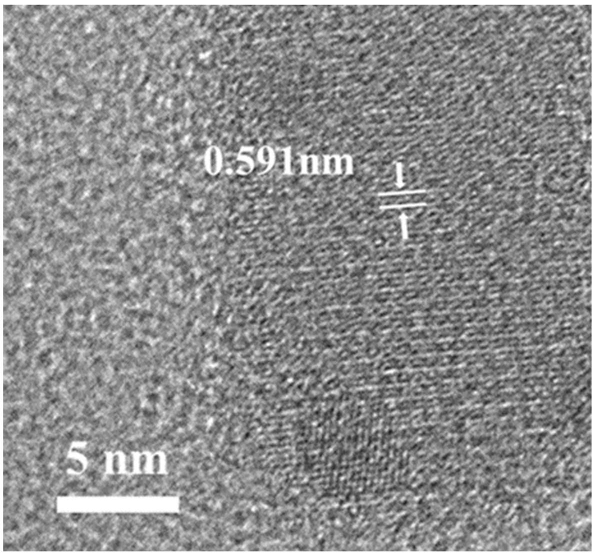

Figure S3. HRTEM images of $CsPbBr_3$ NSs

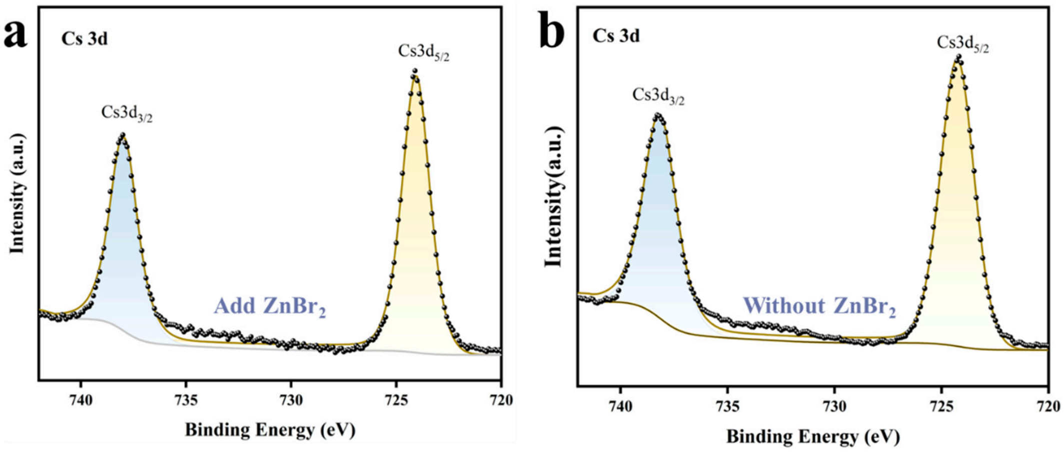

Figure S4. XPS profiles of $CsPbBr_3$ NSs Cs 3d **(a)** with $ZnBr_2$ and **(b)** without $ZnBr_2$

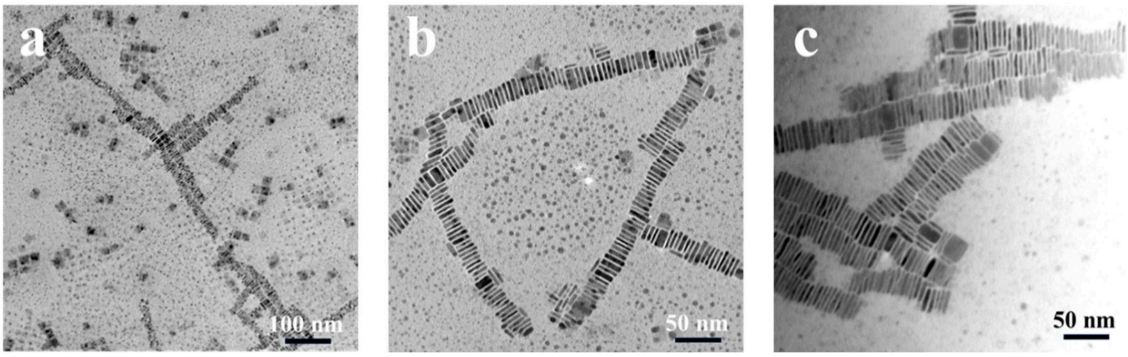

Figure S5. TEM images of CsPbBr3 NSs with transverse dimensions of **(a)** 15nm, **(b)** 22nm, and **(c)** 47nm after self-assembly

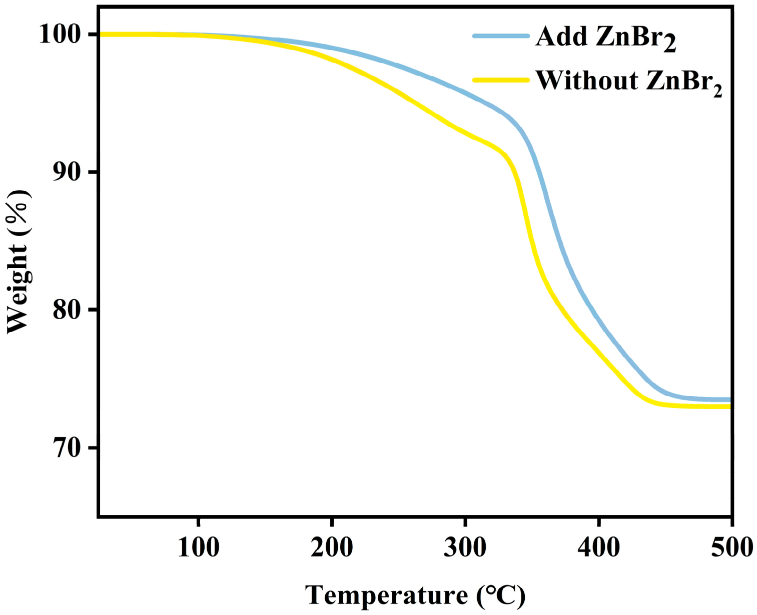

Figure S6. TGA profiles of $CsPbBr_3$ NSs with and without $ZnBr_2$ under N_2 flow and atmospheric pressure in the temperature range 25-500°C

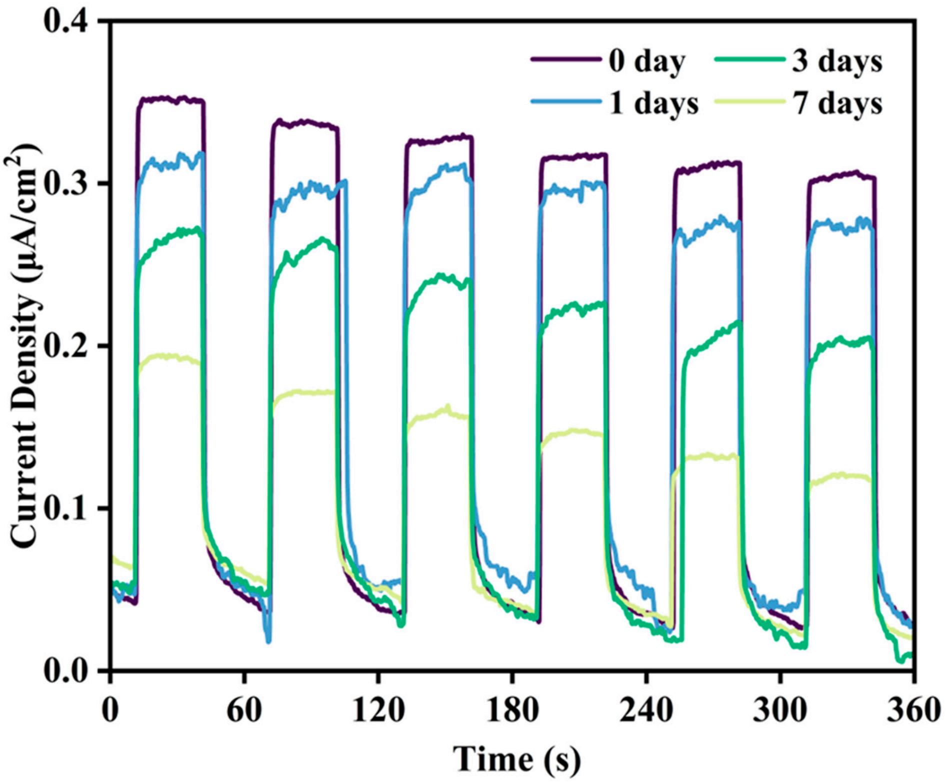

Figure S7. Instantaneous photocurrent response of $CsPbBr_3$ NSs stored at room temperature for 0, 1, 3, and 7 days in neutral water (0.5 M Na_2SO_4) at -0.1 V vs. NHE.
